# Editorial for the Special Issue: Bacterial Meningitis—Epidemiology and Vaccination

**DOI:** 10.3390/microorganisms9050917

**Published:** 2021-04-24

**Authors:** James M. Stuart

**Affiliations:** Population Health Sciences, Bristol Medical School, University of Bristol, Bristol BS8 1QU, UK; james.stuart@bristol.ac.uk

Bacterial meningitis has serious health, economic, and social consequences with a high risk of death and lifelong disability. WHO has published the first global road map on meningitis “Defeating meningitis by 2030” to tackle the main causes of acute bacterial meningitis: *Neisseria meningitidis, Streptococcus pneumoniae, Haemophilus influenzae*, and *Streptococcus agalactiae* (Group B *Streptococcus* (GBS)) [1,2]. The road map was endorsed by the World Health Assembly in November 2020 [3].

The three main goals of the meningitis roadmap are to eliminate epidemics of bacterial meningitis, reduce cases and deaths from vaccine-preventable bacterial meningitis, and reduce disability and improve quality of life after meningitis of any cause. Proposed measures to achieve these goals include development of new vaccines, increased effectiveness of prevention and control strategies, efficient global surveillance with accurate estimates of disease burden including sequelae, and global availability of and access to rapid diagnosis and high-quality treatment of meningitis and its after-effects.

This Special Issue includes a wide range of original research articles and review articles on epidemiology and vaccination of bacterial meningitis that have direct relevance to advancing the goals of the road map.

A fundamental step in establishing the importance of meningitis and in monitoring progress toward prevention and care is quantifying the burden from illness, death, and disability. Wright et al. [4] described wide variation in different modelled estimates of the global burden and advocated for alignment with improving surveillance data to improve the accuracy of model parameters. The consequences of meningitis are even harder to measure. Schiess et al. [5] underlined the social and economic costs of meningitis, the lack of recognition of more subtle sequelae, and the lack of knowledge on long-term effects, especially in low- and middle-income countries. Building care services for those affected by meningitis across the world will be a challenging objective for the meningitis strategy.

The principal means of achieving targets to reduce cases and deaths from meningitis will inevitably be through vaccination. Alderson et al. [6] gave a comprehensive overview of past and present developments in meningitis vaccines. They drew attention to the importance of low-cost vaccines for global introduction, the expanding range of conjugate vaccines and the more recently developed meningococcal protein vaccines, and the challenges in reaching prevention goals. As vaccines are developed and vaccination programmes expanded, Deghmane and Taha [7] made the case that preventing disease among those at higher risk will become increasingly important.

For meningococcal meningitis, the high-burden region of the meningitis belt in sub-Saharan Africa deserves particular attention. Karachaliou Prasinou et al. [8] showed how mathematical models can be used to optimise the effectiveness of vaccination programmes, with two key parameters being the duration of protection and age at vaccination. Such models are relevant both for the serogroup A vaccine currently being deployed in the meningitis belt and for the anticipated roll out of pentavalent (ACWXY) conjugate vaccines [6]. The need for broader-valency vaccines in the global control of invasive meningococcal disease was well demonstrated in the paper by Tzeng and Stephens [9], describing the changing epidemiology and emerging disease due to serogroups other than A, B, and C.

Slack [10] documented how meningitis due to *Haemophilus influenzae* fell rapidly with the introduction of conjugate Hib vaccines from the 1990s such that, by 2015, the burden of meningitis due to serotype b was limited to a few countries that had not introduced these vaccines into the national immunization programmes. However, as she pointed out, invasive disease due to non-typable organisms and other serotypes is of increasing concern. Serotype A has emerged as a significant cause of meningitis in indigenous populations of North America and has stimulated the development of a new conjugate vaccine.

A series of papers from the impressive PSERENADE project [11,12,13] demonstrated the substantial global impact of multivalent pneumococcal conjugate vaccines on invasive pneumococcal disease, including meningitis, after their introduction into infant immunisation programmes. Six or more years after introduction, they found a 95% reduction in serotype 1 disease in all age groups. Measuring the impact does depend on robust serotype surveillance systems, and they acknowledged the need for more data from the meningitis belt countries that are at high risk of pneumococcal meningitis and serotype 1 outbreaks.

Vaccines are in development but not yet available to protect against disease due to GBS [6]. Prevention of GBS in newborns currently relies on risk-based or microbiological screening for infection in pregnancy. However, a study of meningitis among infants under 90 days of age in a large paediatric hospital in the USA [14] showed that the majority of cases of bacterial meningitis were due to GBS, despite universal screening and intrapartum prophylaxis. This only emphasises the importance of a vaccine that could hopefully have more impact than prepartum screening with the additional protection of stillbirths due to GBS infection and late-onset GBS disease.

Tsang [15] focused on the molecular epidemiology of the four main bacterial causes of meningitis in the roadmap and the power of conjugate vaccines to both reduce the burden and drive the evolution of these bacteria, thus underlining the need for improved surveillance and expansion of whole-genome sequencing.

Zainel et al. [16] highlighted neurological complications from bacterial meningitis in children such as hearing loss, cognitive impairment, and epilepsy, as well as the importance of prompt effective treatment regimens in improving outcomes. A key component of prompt treatment is rapid accurate diagnosis of meningitis through bedside tests that can be applicable in low- and middle-income countries. Rondy et al. [17] reported on a field evaluation of a rapid test that should aid timely decisions on vaccine deployment in meningitis epidemics.

Meningitis can be caused by many infectious organisms: bacteria, viruses, fungi, and parasites. The focus in the “Defeating meningitis by 2030” strategy is on the main bacteria responsible for the overall global burden with potential for prevention by vaccination. Another major cause of bacterial meningitis, *Mycobacterium tuberculosis*, was given prominence in this issue by Basu-Roy et al. [18]. Their review highlighted how the “Defeating meningitis” roadmap can be applied to the prevention and control of tuberculosis in children, affirming the need for a collaborative endeavour and linking with activities of other initiatives such as the WHO TB roadmap [19]. The fact that many elements of the roadmap apply to TB and all other causes of meningitis must not be forgotten in the drive to defeat meningitis.

The global roadmap to defeat meningitis is an ambitious strategy, particularly in the context of the Covid pandemic. As shown by the contributions to this Special Issue, a concerted drive to reduce the burden of this illness is, without question, a worthy ambition. The theme of World Meningitis Day 2021 is “Take Action #DefeatMeningitis” [20,21]. Start now!
